# Expression of ArfGAP3 in Vaginal Anterior Wall of Patients With Pelvic Floor Organ Prolapse in Pelvic Organ Prolapse and Non–Pelvic Organ Prolapse Patients

**DOI:** 10.1097/SPV.0000000000000808

**Published:** 2020-04-22

**Authors:** Yu Sun, Bingshu Li, Danhua Lu, Cheng Liu, Shasha Hong, Li Hong

**Affiliations:** From the Department of Gynaecology and Obstetrics, Renmin Hospital, Wuhan University, Wuhan, Hubei, PR China.

**Keywords:** ArfGAP3, pelvic organ prolapse, anterior vaginal wall, RNA expression, protein expression

## Abstract

The purpose of this study was to study the expression of adenosine diphosphate ribosylation factor GTPase-activating protein 3 (ArfGAP3) in the anterior vaginal wall of patients with pelvic organ prolapse (POP).

**Materials and Methods:**

From July 2016 to July 2018, the anterior vaginal wall of 31 POP patients (pelvic organ prolapse quantification [POP-Q] II-III [n = 10] and POP-Q IV [n = 21]) with pelvic floor dysfunction-related symptoms who underwent vaginal hysterectomy were enrolled in POP group in the Department of Gynecology of Wuhan University People’s Hospital. The anterior vaginal wall of 28 non-POP patients who underwent vaginal hysterectomy was selected as control group. The expression of 3 groups was determined by immunohistochemical staining, Western blotting, and quantitative real-time fluorescence polymerase chain reaction.

**Results:**

The expression levels of ArfGAP3 of POP-Q II-III and POP-Q IV groups were lower than the control group (*P* < 0.05), and there were significant differences between POP-Q II-III and POP-Q IV groups (*P* < 0.05).

**Conclusions:**

The expression of ArfGAP3 in the anterior vaginal wall of POP patients decreased, which was related to the pathogenesis and clinical grading of POP.

Pelvic organ prolapse (POP) is a type of pelvic floor support tissue caused by various reasons, resulting in abnormal position and function of organs caused by the decline of pelvic organs. The incidence of POP among women who give birth via vagina worldwide is 40%.^1^ About 50% of parturients have anatomical POP signs. Most of them are asymptomatic. However, with the increase of age and the decrease of postmenopausal estrogen, the weakening of pelvic floor tissue support leads to the occurrence of prolapse-related symptoms, which seriously affects women’s physical and mental health and quality of life.^2^ Epidemiological analysis showed that the pathogenesis of POP was mainly related to childbirth injury, obesity, chronic abdominal pressure increase, age, and menopause, but the specific molecular mechanism was not fully clear.^3^ It is believed that the change of calcium channel can affect the proliferation of fibroblasts and the synthesis, secretion, and degradation of extracellular matrix (ECM), resulting in the decrease of collagen content, imbalance of proportion, and disorder of collagen arrangement in pelvic floor connective tissue, leading to the weakness of pelvic floor supporting tissue and POP.^4,5^ Adenosine diphosphate ribosylation factor GTPase-activating protein (ARF-GAP) family is a diverse protein family with a common ArfGAP domain. The binding of Ca^2+^ with ARF-GAP family proteins can promote the rapid hydrolysis of GTP and play an important role in intracellular vesicle transport.^6^ Studies have shown that, during vesicle transport, calcium channel–dependent synaptic transmission dysfunction can lead to the reduction of axonal release neurotransmitters. More and more evidences show that pelvic floor nerve injury is an important link in the occurrence and development of POP, while abnormal secretion of neurotransmitters and neuropeptides exists in denervated neuropathic tissues.^7,8^ Thus, ARF-GAP family may play an important role in the pathogenesis of POP. In recent years, several proteins with ARF-GAP activity have been identified, but no proteins with ARF-GAP activity have been found in human tissues. The adenosine diphosphate-ribosylation factor GTPase-activating protein 3 (ArfGAP3) is a novel gene found in the 22-week-old human fetal liver DNA library.^9^ Therefore, in this study, we aimed to investigate the expression of ArfGAP3 in anterior vaginal wall of POP patients at different levels and explore its role in the occurrence and development of POP, to provide clues for the etiology of POP.

## MATERIALS AND METHODS

### Clinical Data

From July 2016 to July 2018, the anterior vaginal wall of 31 patients with pelvic floor dysfunction–related symptoms who underwent vaginal hysterectomy for POP were enrolled in POP group. According to the International Continence Society pelvic organ prolapse quantification (POP-Q) score^10^ (Table 1). Stress urinary incontinence was excluded by urodynamic examination and divided into POP-Q II-III group (n = 10) and POP-Q IV group (21 cases). The anterior vaginal wall of 28 non-POP patients who underwent vaginal hysterectomy in our hospital was selected as control group. The POP group and the control group were both married and menopausal, and there were no other connective tissue diseases, pelvic surgery history, or acute or chronic pelvic inflammatory disease history. No estrogens were taken within 3 months before operation. Postoperative pathology confirmed no estrogen-related diseases. There were no significant differences in age, parity, body mass index (BMI), and menopausal status between the groups (*P* > 0.05). (The procedure followed in this study conforms to the ethical standards established by the Human Testing Committee of our hospital and is approved by the Committee.)

**TABLE 1:** POP-Q Stage

Degree	Standard
Stage 0	Point Aa, Ap, Ba, and Bp are all at .3 cm point, and C and D are high at least as high as the vaginal length -tvl~-(tvl-2)cm.
Stage I	The presenting part is >1 cm above the hymen.
Stage II	The leading edge is ≤1 cm above or below the hymen.
Stage III	The leading edge is >1 cm beyond the hymen but ≤1 cm less than tvl.
Stage IV	Complete eversion

### Methods

#### Main Reagent

The following were used in this study: bicinchonininc acid protein assay kit (Pierce Company); enhanced chemiluminescent (Pierce Company); phenylmethylsulfonyl fluoride and radio immunoprecipitation assay tissue lysate (China Wuhan Google Biology Co, Ltd); Trizol kit (Invitrogen); RT-PCR Kit, SYBR Premix ExTaq System (Takara, Japan); polymerase chain reaction (PCR) primers (Shanghai Bioengineering Co, Ltd); biospectrophotometer for Thermo products; Applied Biosystems Plus One qRT-PCR (GeneCopoeia Company); ARFGAP3 antibody (Santa Cruz); GAPDH antibody 1 (Santa Cruz); fluorescent antibody 2 (LI-COR); diaminobenzidine (DAB) chromogenic reagent and streptavidin-perosidase (SP) kit of immunohistochemical *Streptomyces* (Shanjinqiao Biotechnology Co, Ltd); and ordinary inverted microscope and fluorescent positive microscope (Olympus Company of Japan).

#### Draw Materials

The whole anterior vaginal wall of each group was obtained at 12 points of vaginal fornix about 1.0 cm × 1.0 cm in size. After washing with saline, it was dipped in sterile gauze. The tissue was divided into 2 equal pieces, and 1 tissue was immobilized immediately in 4% polyformaldehyde solution for immunohistochemical staining. Another was immediately frozen in liquid nitrogen for Western blot and quantitative real-time fluorescence polymerase chain reaction (qRT-PCR).

#### Immunohistochemical Method

Slices were dewaxed and fed into water. According to the antibody instructions, the phosphate buffered saline (PBS) was repaired by heating acid/hot alkali. The PBS was washed for 5 minutes (2 times). Breeding with 3% H2O2 for 10 minutes and then oxidizing with PBS for 3 minutes were performed. Goat serum was sealed at room temperature for 10 minutes. The blocking solution was carefully inhaled, and the first antibody was added (the goat serum was diluted at 1:100, and the goat serum without the first antibody was negative control). PBS was washed 5 minutes (3 times) after overnight incubation in a wet box. The second antibody was incubated at room temperature for 3 minutes, and PBS was washed for 3 minutes (3 times). The staining was performed with DAB solution and confirmed under microscope, and the washing was terminated. After hematoxylin redyeing and PBS antiblue, tap water was washed and neutral resin seals were made. Positive microscopy was used to observe and take pictures. The results were interpreted according to the comprehensive score of positive staining rate. The experiment was repeated 3 times independently.

#### Western Blot for Protein Detection

After weighing the anterior vaginal wall tissue, the tissue powder was collected by liquid nitrogen milling. The radio immunoprecipitation assay tissue lysate was added to the tissue powder. The tissue was lysed on ice for 30 minutes, 12,000*g*, and 42 centrifugation for 5 minutes using cell ultrasound breaker. The supernatant was transferred to a new eppendorf tube. The total protein concentration was detected by bicinchonininc acid method. The protein was denatured by adding protein sample buffer. The total protein samples of 30 g per hole were separated by 10% polyacrylamide gel electrophoresis gel electrophoresis and transferred to polyvinylidene fluoride membrane; 5% skim milk powder was closed to 1 to 2 hours, 4°C (1:1000), and was incubated overnight; tris-buffered saline tween-20 (TBST) was washed 3 times (5–10 minutes); 10% mg/kg gel electrophoresis was separated by electrophoretic transfer to the gels; and 5% skim milk powder was closed, 4 degrees (1:1000). After overnight incubation, TBST was washed 3 times (5–10 minutes each time). Fluorescent antibody (1:1000) was incubated at room temperature for 1 hour, glyceraldehyde-3-phosphate dehydrogenase (GAPDH) (1:1000) was used as internal reference, and TBST was washed 3 times (10 minutes each time) and then scanned by Odyssey dual-color infrared laser imaging system. With GAPDH as the internal reference, the gray ratio of Western bands was calculated by Quantity One (version 4.6.2) software as the relative expression level of each target protein. The experiment was repeated 3 times independently.

#### Quantitative Real-Time Fluorescence PCR

Total RNA was extracted by Trizol method, and 1 μg of total RNA was added into 20 μl reaction system to synthesize DNA according to the instructions. Real-time PCR was performed on Applied Biosystems 7500 quantitative real-time PCR. The GAPDH was used as internal reference, and ArfGAP3 as target gene. The sequence of primers is shown in Table 2. The amplification conditions were as follows: 95°C, 30 seconds → 95°C, 5 seconds → 60°C, 34 seconds → 95°C, 15 seconds → 60°C, 1 minute → 95°C, 15 seconds → 60°C, 15 seconds, 40 cycles. The experiment was repeated 3 times independently.

**TABLE 2:** Primer Sequences Used for RT-PCR

Gene	Oligonucleotide Sequence
GAPDH	F 5′-AGGAGCGACCCCACTAACA-3′ R 5′-AGGGGGGCTAAGCAGTTGGT-3′
ARFGAP3	F 5′-CGAGGAGGAAGTACCAGGAGGAC-3′, R 5′-CCAGTAGGAATCGGCACCATCATC-3′

#### Result Criteria

##### Immunohistochemical

Under the microscope, 5 high-power visual fields were randomly selected from each slice for observation. The positive staining of ArfGAP3 was brown and yellow. The expression of ArfGAP3 was mainly concentrated in lamina propria and a few in mucosa. The positive expression was determined when the staining intensity was significantly higher than the background. Positive cells were classified into 4 grades according to their number and color intensity. No positive cell expression was determined as (−). The number of positive cells was less than 10%, and the staining intensity that was weak positive was determined as weak positive (+). The number of positive cells was more than 60%, and a few cells that were weakly positive was determined as strong positive (+++). The number and intensity of positive cells that were between weak positive and strong positive were determined as positive (++). The positive rate of ArfGAP3 expression was calculated as follows: the number of specimens per group (+)~(+++)/the total number of specimens per group.

##### Western Blot

The expression level of ArfGAP3 was calculated as follows: (ArfGAP3 band gray value/GAPDH band gray value) × 100%.

### Statistical Methods

The experimental data were analyzed by SPSS19.0 statistical analysis software, the measurement data were statistically described by mean ± standard deviation, and the differences between groups were analyzed by single factor variance analysis. Multiple There were significant differences in age, menopausal status, BMI, and labor between the control group and the POP group (Table 3).

**TABLE 3:** Sociodemographic Characteristics Findings for Cases and Controls

Characteristics	n	Age, y	Menopausal, y	BMI, kg/m^2^	Vaginal Party
POP-Q II-III	10	62.83 ± 7.58	12.08 ± 8.90	24.79 ± 2.43	1.99 ± 1.27
POP-Q IV	21	62.23 ± 7.93	12.20 ± 7.64	22.36 ± 1.86	2.16 ± 1.64
Control	28	61.84 ± 6.94	11.03 ± 9.61	23.72 ± 2.59	2.37 ± 1.58
*P*		0.186	0.570	0.600	0.733

Data are presented as mean ± SD.

### Immunohistochemical Detection of ArfGAP3 Expression in Anterior Vaginal Wall

**FIGURE 1:**
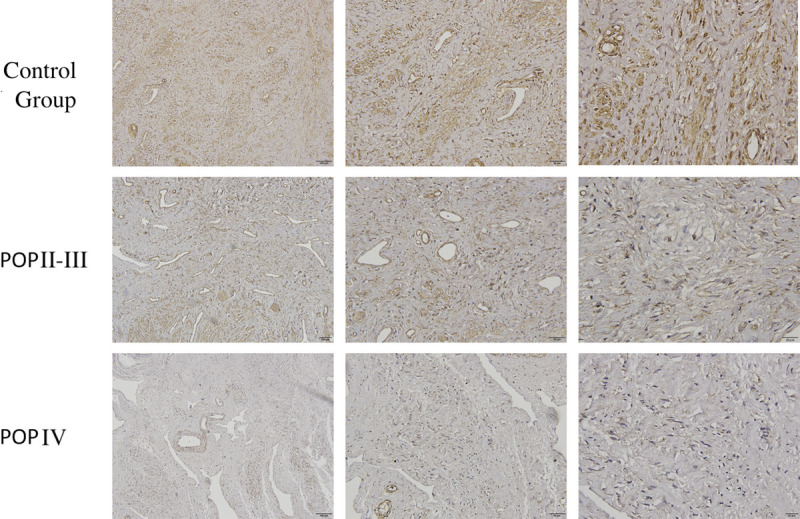
Immunohistochemica analysis of ArfGAP3 proteins detected in anterior vaginal wall with POP (SP, ×40, ×100, and ×200).

Immunohistochemical results showed that ArfGAP3 was expressed in both the control group and POP group, mainly in the lamina propria. According to the experimental results, a large number of dark yellow positive particles appeared in the control group, and the distribution was dense, significantly higher than the background color. Positive staining in POP group (POP-Q II-III, POP-Q IV) was pale yellow with low expression and sparse distribution. With the increase of prolapse degree, the expression of ArfGAP3 in anterior vaginal wall tissue of POP group and control group was less. There was significant difference in ArfGAP3 expression between POP group and control group (*P* < 0.05). In POP-Q II-III group, ArfGAP3 was positive in 7 of 10 cases, of which (+) was positive in 5 cases and (++) was positive in 2 cases. The total positive expression rate was 70%. In POP-Q IV group, 6 of 21 cases were (+) positive, and the total positive rate was 28.6%. Among 28 cases of control group, 27 were positive for ArfGAP3, of which 2 were positive for (+), 3 were positive for (++), and 22 were positive for (++). The total positive rate was 96.4% (Table 4, Fig. 1). Compared with the control group, POP group had statistical significance (*P* < 0.05).

**TABLE 4:** Positive Expression of ArfGAP3 in Each Group

Group	n	−	+	++	+++	Positive Rate, %
POP-Q II-III	10	3	5	2	0	70 (7/10)
POP-Q IV	21	15	6	0	0	28.6 (6/21)
Control	28	1	2	3	22	96.4 (27/28)
*P*						0.001

### Western Blotting Detection of ArfGAP3 Expression in Anterior Vaginal Wall

**FIGURE 2:**
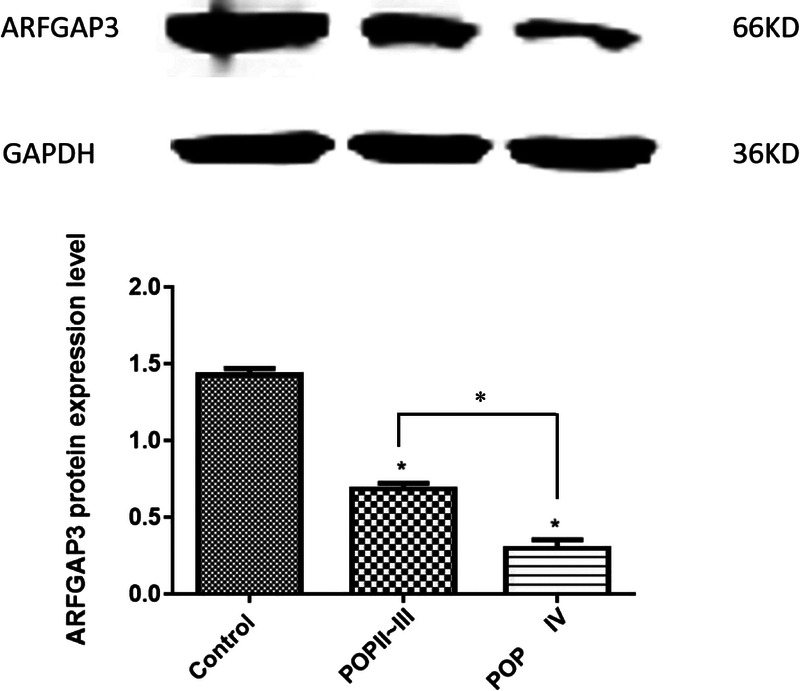
Representative Western immunoblot and densitometric analysis of ArfGAP3 proteins detected in anterior vaginal wall with POP. The results shown are the mean ± SEM. A statistically significant difference is indicated by *P < 0.05.

The results of Western blot analysis showed that the protein expression level of ArfGAP3 decreased gradually in the control group, the POP-Q II-III group, and the POP-Q group (Fig. 2). The expression of ArfGAP3 between the 3 groups was statistically significant (*P* = 0.000). The expression of ArfGAP3 in the control group was significantly higher than that of the POP-Q II-III group and the POP-Q IV group (*P* = 0.000), and the POP-Q II-III group and the POP-Q group were statistically significant (*P* = 0.000) (Table 5).

**TABLE 5:** Comparison of Western Blotting Analysis of Expression of ArfGAP3 in All Groups

Group	n	Expression of ArfGAP3
POP-Q II-III^。^	10	0.694 ± 0.035
POP-Q IV^。^	21	0.375 ± 0.047
Control	28	1.486 ± 0.072
*P*		0.000

### qRT-PCR Detection of ArfGAP3 Messenger RNA Expression in Anterior Vaginal Wall

**FIGURE 3:**
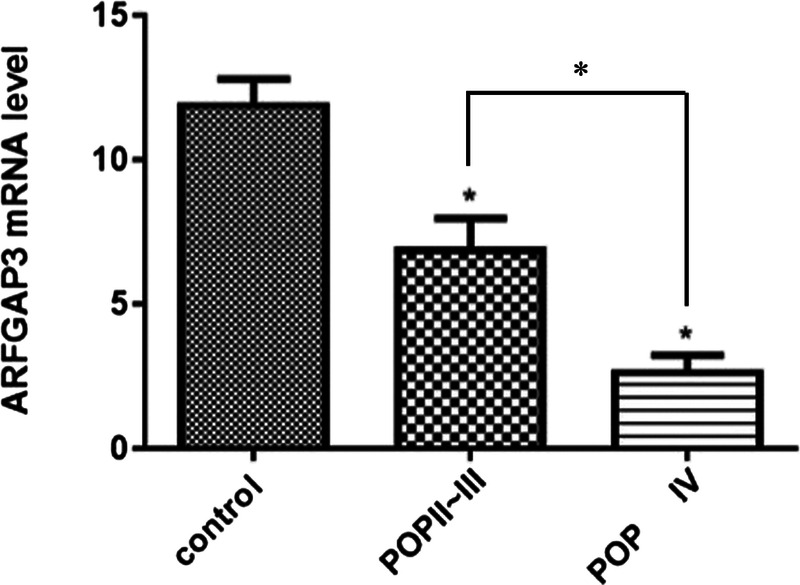
The qRT-PCR analyses of ArfGAP3 messenger RNA levels in anterior vaginal wall from women with control, POP II-III, and POP IV groups. The results are shown the mean ± SEM. A statistically significant difference is indicated by *P < 0.05.

The results of qRT-PCR showed that the protein expression level of ArfGAP3 was decreased in the control group, the POP-Q II-III group, and the POP-Q group (Fig. 3). The expression of ArfGAP3 between the 3 groups was statistically significant (*P* = 0.001). The expression of ArfGAP3 in the control group was significantly higher than that of the POP-Q II-III group and the POP-Q group IV group (*P* = 0.001), and the POP-Q II-III group and the POP-Q group were statistically significant (*P* = 0.001) (Table 6).

**TABLE 6:** Comparison of Messenger RNA Relative Expression of ArfGAP3 in All Group

Group	n	Expression of ArfGAP3
POP-Q II-III^。^	10	6.71 ± 0.82
POP-Q IV^。^	21	2.61 ± 0.51
Control	28	11.85 ± 0.92
*P*		0.001

## DISCUSSION

Pelvic organ prolapse is a kind of pelvic floor tissue degeneration, trauma, congenital dysplasia, or injury caused by some diseases and weakening of tension, resulting in the weakening of its supporting function and the downward displacement of female reproductive organs and adjacent organs, including vaginal anterior and posterior wall prolapse, uterine prolapse, and rectal bladder prolapse. Its risk factors include pregnancy injury and vaginal delivery, menopause, and long-term abdominal pressure increase.^11^ With the increase of age, the prevalence rate is increasing, which affects not only the health of patients but also the mental health and quality of life of patients in various degree. It has become a global medical and public health problem. At present, the mechanism of POP is generally acknowledged as the whole pelvic floor theory and the hammock theory; therefore, the occurrence of POP is related to the change of the function of the pelvic floor supporting structure. It is speculated that the defect of the anterior vaginal wall leads to the weakening of the pelvic floor supporting structure, which may lead to the occurrence of POP. Therefore, the anterior vaginal wall tissue was selected as the sample site in this study.^12^ It has been found that abnormal peptide neurotransmitters and pelvic floor nerve injury mechanism are important links in the occurrence and development of POP. Busacchi et al^13^ demonstrated the existence of neurotransmitter-synthesizing neurons in the pubococcygeal muscle around the rectum. Neuropeptide Y and vasoactive intestinal peptide released by neurotransmitters were found to decrease in the levator ani muscle and periurethral muscle of patients with severe POP. North et al^14^ compared the sensory function of pelvic floor tissue between POP patients and healthy women. The results showed that the sensory threshold of vagina and clitoris increased in POP patients, whereas that of healthy women was within the normal range, which confirmed that the sensory and motor function of pelvic floor nerve in POP patients were decreased. ArfGAP3 is a GTPase activator protein that participates in vesicle transport pathway and mediates neurotransmitter release.^15^ Therefore, we speculate that the abnormal changes of vesicle transport mediated by ArfGAP3 may be involved in the occurrence of POP. Abnormal vesicle transport has been extensively studied in the field of neurological diseases. Hui-Yun et al’s^16^ model of Alzheimer’s disease observed a large number of vesicles missing at the synaptic end of neurons and that mitochondrial transport slowed down or stagnated, which confirmed that abnormal vesicle transport promoted the occurrence and development of Alzheimer’s disease. At present, there is no relevant report about abnormal vesicle transport in POP disease. Therefore, this study aimed at the expression of ArfGAP3 in the anterior vaginal wall of POP patients to explore the role of ArfGAP3 in the occurrence and development of POP and provide reference for clinical diagnosis and treatment.

The ARF-GAP belongs to the Ras superfamily of GTP-binding proteins, including the ArfGAP1 subfamily, the ArfGAP2 subfamily, the adhesion and degranulation-promoting adapter protein subfamily, the small molecule activators of protein subfamily, the Arf GTPase-activating proteins subfamily, the Arf GAP domain and FG repeats containing protein subfamily, and the Australian Standardised Affordability and Pricing subfamily, among others. Similar to the Ras protein, ARF and GTP binding is active and is an important intracellular substance transport regulator.^17^ The ArfGAP can catalyze ATP hydrolysis by combining with cytoskeleton-related molecular motors, such as actin and myosin, and release energy to generate power to promote vesicle transport.^18^ Studies have confirmed that it promotes vesicle transport by inducing the formation of F-actin folds.^19^

In this study, 3 experimental methods were used to detect the expression of ArfGAP3 in the anterior vaginal wall of POP group and control group. We found that ArfGAP3 was downregulated in POP group and negatively correlated with the severity of POP. Therefore, we concluded that the decrease of ArfGAP3 in the anterior vaginal wall may be closely related to POP. Recent studies have shown that ARF-GAP family protein has Ca^2+^-dependent domain and the Ca^2+^ is combined with the domains to stimulate the rapid hydrolysis of GTP and participates in the regulation of intracellular and extracellular vesicle transport. Researchers used brefeldin A to block the binding of ARF and GTP, when after interfering with the activity of intracellular ARF, they found that the manipulative calcium influx was downregulated, suggesting that the binding of Ca^2+^ to ARF-GAP family proteins played a crucial role in mediating vesicle transport and promoting the release of neurotransmitters.^20^ The present studies have shown that abnormal condition of Ca^2 +^ secretion is associated with pelvic floor dysfunction and neuroendocrine diseases. Through the study of hepatic stellate cells, it was found that the synthesis of collagen I required a very high intracellular Ca^2+^ concentration, and the inactivation of intracellular calcium pump significantly reduced the synthesis of collagen I, which indicated that the change of Ca^2+^ could affect the synthesis of ECM.^21^ Min et al^22^ found that electrical stimulation could induce the opening of T-type calcium channel, change the intracellular Ca^2+^ concentration, then activate the TGF-β1-Smad2/3 pathway, induce the deposition of ECM, and achieve the therapeutic effect on stress urinary incontinence. Huynh and Vale^23^ found that abnormal Ca^2+^ secretion could induce abnormal transport pathways in axons of motor neurons regulated by kinesin, which affected the release of neurotransmitters and eventually led to motor neuron disease in mice. Gauthier and Wollheim’s^24^ study on the pathogenesis of diabetes mellitus found that the amount of insulin released by pancreatic β cells depended on the exocytosis of protein granules secreted by pancreatic β cells in the calcium-dependent pathway. If the secretion of Ca^2+^ was abnormal, the vesicle transport was abnormal and leads to a decrease in the secretion of insulin. ArfGAP3 is a GTPase-activated protein that regulates retrograde transport from Golgi to endoplasmic reticulum. Like other members of the ARF-GAP family, ArfGAP3 contains a unique GATA-1 zinc finger motif. It exhibits strong GAP activity to ARF1 protein, which can dissociate vesicles from Golgi and promote vesicle transport.^9^ Synaptotagmin can transmit calcium signals to synaptic vesicles quickly and accurately during vesicle transport. By acting with soluble NSF attachment protein receptor complex, it can fuse with cell membranes and release neurotransmitters, and complete the transmission of nerve information. Therefore, Ca^2+^ plays an important role in vesicle transport.^25^ As a ubiquitous second messenger in cells, Ca^2+^ plays an important role in many physiological and pathological processes of cells, so the imbalance of Ca^2+^ homeostasis will lead to related diseases inevitably. In this study, we preliminarily believe that the downregulation of ArfGAP3 gene level in pelvic floor supports tissues of POP patients and the inhibition of vesicle transport function and that the downregulation of manipulative calcium influx in calcium pool causes abnormal metabolism of peptide neurotransmitters and ECM, thus participating in the occurrence and development of POP. With the decrease of ArfGAP3 expression, POP grading tends to increase.

In recent years, with the trend of population aging, the incidence of POP disease is increasing year by year. Although the pathogenesis is still uncertain, we found that the downregulation of ArfGAP3 gene in anterior vaginal wall may play an important role in the pathogenesis and development of POP. Therefore, we can further study the related pathways through the research basis of this experiment, which can provide new ways and help for the future diagnosis and treatment of POP.
